# AI-Powered Predictive Models in Implant Dentistry: Planning, Risk Assessment, and Outcomes

**DOI:** 10.3390/jcm15010228

**Published:** 2025-12-27

**Authors:** Ghada Neji, Roberta Gasparro, Mohamed Tlili, Aya Dhahri, Faten Khanfir, Gilberto Sammartino, Angelo Aliberti, Maria Domenica Campana, Faten Ben Amor

**Affiliations:** 1 Oral and Facial Rehabilitation Research Laboratory, Faculty of Dentistry, University of Monastir, Monastir 5000, Tunisia; nejighadaa96@yahoo.fr (G.N.); drtlilimohamed@gmail.com (M.T.); aya.dhahri@yahoo.fr (A.D.); khanfirfaten@yahoo.fr (F.K.); faten.benamor@yahoo.fr (F.B.A.); 2 Department of Neuroscience, Reproductive Science and Dentistry, University of Naples Federico II, 80131 Naples, Italy; roberta.gasparro@unina.it (R.G.); mariadomenica.campana@gmail.com (M.D.C.)

**Keywords:** artificial intelligence, implant dentistry, implant planning, implant navigation

## Abstract

Artificial intelligence (AI) is rapidly transforming the landscape of dental implantology by enhancing every stage of treatment, from diagnostics and digital planning to intraoperative navigation, outcome prediction, and long-term follow-up. This narrative review explores the current and emerging applications of AI technologies in implant dentistry, with a focus on machine learning, neural networks, and computer vision. It examines how AI is utilized in digital implant planning, surgical navigation, peri-implant disease monitoring, risk assessment, and the prediction of treatment outcomes such as peri-implantitis and implant failure. These innovations contribute to more efficient workflows, more personalized treatment strategies, and improved cost-effectiveness of care. Finally, future perspectives and educational implications of AI integration in clinical implantology are discussed.

## 1. Introduction

Over recent years, digital dentistry has undergone significant advancements, leading to a profound transformation in diagnostic, planning, and therapeutic workflows. Innovations include low-dose, high-resolution cone-beam computed tomography (CBCT), intraoral scanners (IOS), computer-aided design and manufacturing (CAD/CAM) systems, three-dimensional (3D) medical printing technologies, and dynamic navigation systems [1]. While the digital workflow is applicable across various domains of dentistry and dento-maxillofacial practice, its most sophisticated applications are currently observed in the fields of implantology and restorative dentistry [2]. Among these technological developments, artificial intelligence (AI) has emerged as a transformative tool, increasingly integrated into multiple phases of dental treatment planning and execution. In implant dentistry, AI is facilitating a paradigm shift in clinical decision-making by enhancing the accuracy of planning, enabling more robust risk assessments, and improving the prediction of treatment outcomes [3,4]. Through the analysis of large-scale patient data, AI enables clinicians to make more accurate and individualized decisions, ultimately improving treatment success and reducing complications.

Unlike existing narrative and systematic reviews that primarily focus on cataloging artificial intelligence applications or reporting diagnostic accuracy, the present review adopts a clinically oriented perspective by integrating diagnostic, prognostic, and surgical AI models within unified clinical decision-making pathways. Emphasis is placed on model calibration, interpretability, validation status, and real-world clinical utility, with the aim of bridging the gap between technical performance and responsible clinical implementation.

## 2. Literature Search

This narrative review was designed to provide a critical and clinically oriented synthesis of the current literature on artificial intelligence applications in implant dentistry. A structured, non-systematic literature search was conducted using PubMed/MEDLINE, Scopus, and Web of Science databases. The search focused on articles published between January 2015 and December 2024 and was limited to English-language publications. Search terms were combined using Boolean operators and included: “artificial intelligence”, “machine learning”, “deep learning”, “convolutional neural network”, “implant dentistry”, “dental implants”, “peri-implantitis”, “implant failure”, “risk assessment”, “diagnosis”, “navigation”, and “surgical planning”. Eligible articles included narrative and systematic reviews, clinical studies, validation studies, and methodological papers addressing diagnostic, prognostic, and surgical AI models in implant dentistry. Studies were selected based on their relevance to clinical decision-making, model performance, interpretability, and translational applicability, rather than on predefined quantitative inclusion criteria, in accordance with the narrative review framework. Reference lists of key articles were also manually screened to identify additional relevant studies.

## 3. Overview of AI Learning Paradigms Relevant to Implant Dentistry

Artificial intelligence systems applied in implant dentistry rely on different computational paradigms, each characterized by specific advantages and methodological constraints that influence clinical applicability. Supervised learning remains the most widely used approach in diagnostic and prognostic modeling, where algorithms are trained on labeled datasets to predict outcomes such as peri-implant bone loss, implant failure, or the need for sinus augmentation by learning associations between clinical variables and target outputs [5]. These models offer relatively high interpretability and can integrate heterogeneous clinical parameters; however, their performance is strongly dependent on data quality, and small or imbalanced datasets may limit generalizability, particularly in implant dentistry where multicenter data remain limited. Unsupervised learning explores unlabeled datasets to identify latent structures and intrinsic patterns. Although less frequently applied in dental implantology, clustering and dimensionality-reduction techniques may reveal previously unrecognized patient phenotypes, risk profiles, or radiographic patterns, contributing mainly to hypothesis generation rather than direct clinical application due to current validation challenges [6]. Deep learning (DL) represents a major advancement for analyzing complex imaging datasets, enabling automatic extraction of hierarchical features from radiographic or CBCT images without manual feature engineering [7]. This makes DL particularly effective for detecting subtle peri-implant radiographic alterations; however, these models require large, well-annotated datasets, substantial computational resources, and rigorous external validation to ensure clinical reliability. Within dental imaging, convolutional neural networks (CNNs) are the dominant DL architecture and have demonstrated high accuracy in bone segmentation, implant classification, detection of peri-implant pathology, and prediction of osseointegration [8]. Despite their strong diagnostic performance, CNNs are limited by reduced interpretability, black-box behavior, and susceptibility to dataset bias, prompting growing interest in explainable AI techniques, whose application in implant dentistry remains limited [9]. Distinguishing among supervised learning, unsupervised learning, deep learning, and CNN architectures is essential, as these paradigms differ in data requirements, interpretability, and translational potential. Supervised models generally offer greater transparency but lower performance on unstructured imaging data, whereas CNNs provide superior diagnostic accuracy while remaining more difficult to interpret and validate across diverse populations.

## 4. Diagnostic AI Models in Implant Dentistry

Diagnostic AI systems focus on identifying existing peri-implant alterations, including marginal bone loss, peri-implantitis, and early radiographic signs of inflammation. These models operate exclusively within the diagnostic domain, meaning they assess current pathological conditions without attempting to predict future complications.

Convolutional neural networks (CNNs) have become the predominant method for radiographic diagnostic tasks due to their ability to automatically extract hierarchical features from periapical radiographs and CBCT scans. Prior studies demonstrated that CNNs can reliably detect peri-implant bone defects, radiolucencies, and soft-tissue changes associated with early disease stages [10,11]. More recent investigations further confirm that neural networks outperform traditional diagnostic methods in identifying subtle bone level changes and early peri-implantitis on radiographic imaging [12].

By enabling earlier recognition of peri-implant disease, diagnostic AI tools support clinical decision-making and may contribute to reducing the risk of progression to severe peri-implantitis. Importantly, these systems differ conceptually from prognostic models: they do not estimate long-term outcomes or implant survival but provide high-accuracy analysis of the current implant condition.

## 5. Prognostic AI Models for Risk Assessment and Outcome Prediction

### 5.1. Implant Risk Assessment

In implant dentistry, prognostic models play a central role in estimating the probability of specific clinical outcomes, thereby supporting evidence-based decision-making and optimizing patient management. By systematically evaluating individual risk factors such as systemic health, bone quality, and periodontal history, these models enable clinicians to anticipate complications and implement personalized preventive or therapeutic strategies. The integration of artificial intelligence (AI) has markedly enhanced risk assessment by enabling the analysis of large and heterogeneous datasets, including clinical, radiographic, and biological information, to generate individualized risk profiles for implant failure, peri-implant disease, and prosthetic complications [13]. Accurate identification of patients at elevated risk for peri-implantitis is essential for preventing implant loss and ensuring long-term success. Contemporary risk assessment frameworks integrate multiple clinical, biological, behavioral, and systemic domains and have progressively evolved from traditional statistical approaches toward advanced computational techniques, such as machine learning and deep learning, capable of modeling complex non-linear interactions among predictive variables [14]. A recent systematic review demonstrated that AI-based diagnostic and prognostic models can analyze radiographic images to detect early pathological changes, including marginal bone loss and implant instability, achieving high sensitivity and specificity while improving reproducibility and reducing reliance on time-intensive manual assessments [12]. Systemic conditions, particularly diabetes mellitus, have also been incorporated into predictive frameworks to refine risk stratification, given their multifactorial impact on peri-implant tissue health through impaired immune response, delayed wound healing, and increased inflammatory burden [15]. Supporting clinical evidence includes a prospective cohort study by Zhang et al., which identified residual periodontal pockets and implant positioning as significant predictors of peri-implantitis and proposed a clinician-friendly nomogram for individualized risk estimation in patients with a history of severe periodontitis [16]. Beyond static risk stratification, AI-driven tools are increasingly integrated into clinical decision support systems and digital patient monitoring platforms, allowing dynamic updating of risk profiles based on longitudinal data. Continuous analysis of follow-up radiographs, peri-implant tissue parameters, and systemic health indicators enables early detection of disease progression and promotes a shift from reactive to predictive and preventive implant care. The future integration of AI analytics with wearable biosensors and electronic health records may further support personalized and adaptive implant maintenance strategies, improving both clinical outcomes and efficiency [8,17].

### 5.2. Outcome Prediction and Long-Term Success Models

In implant dentistry, the assessment of treatment success has traditionally relied on clinical expertise, radiographic interpretation, and manual planning. Although these approaches have contributed substantially to the development of predictable protocols, they remain subject to variability in operator experience and interpretive bias, which may affect diagnostic consistency and treatment outcomes. As implant procedures become increasingly sophisticated and patient expectations regarding function and esthetics continue to rise, the demand for tools that enhance precision, objectivity, and reproducibility has grown considerably. Artificial intelligence (AI) represents a major technological advancement capable of addressing these challenges by integrating computational precision with clinical reasoning to improve both preoperative assessment and long-term follow-up. The continuous progress of AI technologies and the exponential growth of digital clinical data have enabled the application of neural network–based systems in various aspects of implant dentistry. These systems are designed to recognize complex, non-linear relationships among numerous biological and mechanical variables that influence implant success. Through advanced pattern recognition and feature extraction, neural networks can analyze extensive datasets including patient demographics, systemic conditions, bone quality, implant dimensions, and prosthetic factors to assist clinicians in diagnosis, treatment planning, and prognosis prediction [18,19]. Importantly, these models allow for a shift from experience-based to data-driven decision-making, enhancing reproducibility and supporting a more personalized approach to patient care. Lyakhov et al. developed a neural network system to predict the success of single dental implants, reporting a test accuracy of 94.48%. The model evaluated 55 statistical parameters encompassing systemic health indicators, dentoalveolar status, and site-specific anatomical features. A large database of digitized patient records facilitated the model’s training through multivariate data analysis [20]. Although the authors emphasized that such systems cannot replace clinical judgment, they demonstrated that AI could serve as a valuable diagnostic adjunct to guide clinicians in treatment selection and risk assessment. In a related study, Liu et al. developed a predictive model for the early detection of potential implant rejection based on clinical data from 681 patients and 20 predictive variables. Using supervised learning methods, the model achieved a prediction accuracy of 74.10%, which was approximately 20 percentage points lower than that of the neural network model proposed by Lyakhov et al. [20]. The reduced accuracy was attributed to limitations in data volume and the number of influencing factors incorporated during training [21]. This highlights the critical importance of comprehensive datasets and appropriate variable selection in optimizing model performance and clinical reliability.

Further insights were provided by Oh et al. [22], who evaluated seven distinct deep learning architectures for predicting implant osseointegration. Their systematic comparison of model sensitivity, specificity, and accuracy repeated across ten independent training and validation cycles yielded an average predictive accuracy of approximately 80%. These results underscore the potential of deep learning to enhance diagnostic precision, improve prognostic modeling, and support evidence-based decision-making in implant dentistry [22]. Overall, AI-based predictive models offer a promising framework for estimating implant outcomes and improving long-term clinical success. Nevertheless, several challenges remain, including dependence on the quality and heterogeneity of input data, limited external validation across diverse populations, and the potential for algorithmic bias introduced by unbalanced datasets. Additionally, the interpretability of complex neural networks often described as “black box” systems poses an obstacle to full clinical integration [23]. Future advancements should focus on developing explainable AI (XAI) models capable of providing transparent decision pathways, as well as on creating standardized multicenter databases for model training and validation [24]. Within this framework, AI should be regarded not as a substitute for clinician expertise, but as a complementary tool that augments clinical judgment, supports personalized treatment planning, and contributes to the broader transition toward predictive, preventive, and precision implant dentistry.

### 5.3. Comparative Performance of AI Models: Accuracy, Sensitivity, and Specificity

When synthesizing available evidence across diagnostic and prognostic studies, several consistent trends emerge regarding model performance, data dependency, and validation limitations, regardless of the specific algorithm employed. A direct comparison of accuracy, sensitivity, and specificity across AI models reveals substantial variability influenced by dataset characteristics, model architecture, and validation methods. Traditional machine learning algorithms such as logistic regression, random forests, or support vector machines typically report accuracies ranging from 65% to 80% in predicting implant failure or peri-implant disease, as observed in the supervised models evaluated by Liu et al. 2018 [21]. Their sensitivity tends to be moderate, often below 70%, reflecting limitations in detecting minority pathological cases, especially when datasets are imbalanced. Specificity values are usually higher, indicating stronger performance in identifying healthy or low-risk sites, but this may lead to underestimation of early complications.

Deep learning approaches, particularly neural networks trained on structured clinical data, often demonstrate superior accuracy, frequently exceeding 80–90%, as reported by Lyakhov et al. [20] in models predicting implant survival. However, their sensitivity remains highly dependent on the diversity and representativeness of the training data, with some models performing well on training sets but showing reduced sensitivity during external testing. This discrepancy highlights the persistent challenge of overfitting and poor generalizability, a concern also emphasized in systematic reviews on AI prognostic modeling in dentistry [4].

Convolutional neural networks (CNNs), especially those trained on radiographic or CBCT datasets, consistently achieve the highest performance in imaging-based tasks. Several studies report accuracy values above 90% for detecting peri-implant radiolucency, marginal bone loss, or implant mispositioning, as shown by Oh et al. [22] and in CNN-based diagnostic studies summarized by Alqutaibi et al. [12]. Specificity values are similarly high, confirming the robustness of CNNs in differentiating healthy versus pathological tissues. Sensitivity, while often strong, can vary significantly depending on lesion size, image resolution, network depth, and annotator variability. CNNs demonstrate a clear advantage in identifying subtle radiographic patterns that may not be captured by traditional ML models; however, their dependency on large, annotated datasets limits scalability and standardization.

Despite these promising results, direct comparison across studies remains challenging, as performance metrics are affected by heterogeneous sample sizes, variability in imaging modalities, inconsistent definitions of “ground truth,” and differences in validation protocols. Moreover, high statistical accuracy does not always translate into clinical utility. A model with excellent specificity but suboptimal sensitivity may fail to detect early peri-implant pathology, while a highly sensitive model with lower specificity may lead to overtreatment. These clinical implications align with recent discussions on the need for balanced evaluation metrics and model calibration in dental AI research [25]. Therefore, a comprehensive assessment of accuracy, sensitivity, and specificity is essential when evaluating the real-world applicability of AI systems in implant dentistry.

### 5.4. Interpretability Challenges and the Black-Box Nature of AI Models

A major limitation of many high-performing AI systems in implant dentistry is their limited interpretability. Deep learning models, particularly convolutional neural networks (CNNs), are often described as “black box” architectures because they generate predictions without providing insight into the internal reasoning processes that lead to a specific output. This lack of transparency poses significant concerns for clinical adoption, as dentists and implant surgeons must be able to justify diagnostic and prognostic decisions both clinically and legally. The opacity of these models also complicates error analysis, making it difficult to determine whether incorrect predictions arise from data imbalance, image noise, selection bias, or spurious correlations learned during training [23].

Interpretability is particularly critical in implant dentistry, where treatment planning frequently relies on the assessment of anatomical structures, bone morphology, and prosthetic constraints. If a model predicts high risk for implant failure or peri-implantitis without explaining which radiographic features or clinical variables influenced the prediction, clinicians may be hesitant to modify their treatment decisions based solely on the algorithm’s output. This problem is compounded by the fact that many AI systems are trained on single-center datasets with limited demographic variability, increasing the risk that the model learns site-specific biases rather than generalizable clinical patterns [10].

Recent advances in explainable artificial intelligence (XAI) offer promising strategies to mitigate these challenges. Techniques such as saliency maps, Grad-CAM visualization, occlusion analysis, and feature importance ranking can help identify which image regions or variables contributed most to the model’s decision [26]. These tools may enhance clinician trust, facilitate regulatory approval, and support safer integration of AI into surgical planning and risk assessment workflows. However, their application in dental and implant-related AI research remains limited, and no standardized guidelines currently exist for reporting interpretability analyses in diagnostic or prognostic models [23,24]. Overall, improving the interpretability of AI systems is essential for bridging the gap between technical performance and clinical usability. Transparent, explainable models, combined with robust external validation, represent a crucial step toward ensuring that AI-supported decision-making aligns with ethical, medico-legal, and patient-centered standards in implant dentistry.

### 5.5. Clinical Significance Versus Statistical Performance of AI Models

Although AI models in implant dentistry frequently report high accuracy, sensitivity, or specificity, these statistical indicators do not always translate into meaningful clinical benefit. Performance metrics are often derived from controlled research settings characterized by standardized imaging protocols, curated datasets, and balanced outcome distributions. However, real-world clinical environments present far greater heterogeneity in patient anatomy, systemic health conditions, radiographic quality, and disease presentation, which may significantly diminish model performance once deployed in practice [12,27]. From a clinical perspective, the trade-off between sensitivity and specificity is particularly relevant. A model with excellent specificity but limited sensitivity may fail to detect early peri-implant pathology or subtle signs of implant instability, potentially delaying intervention and increasing the risk of complications. Conversely, highly sensitive models with insufficient specificity may generate excessive false positives, leading to unnecessary follow-up imaging, overtreatment, increased costs, and heightened patient anxiety. This highlights the fact that statistical performance metrics must be interpreted considering their clinical consequences, rather than as isolated numerical indicators [13].

Another key determinant of clinical usefulness is model calibration, which assesses the agreement between predicted risk and the true probability of an outcome. Poorly calibrated prognostic models may systematically overestimate or underestimate peri-implant disease risk, resulting in inappropriate treatment planning. For example, a model that overestimates peri-implantitis risk may prompt unwarranted interventions, while an underestimating model may delay treatment in patients requiring early management. Calibration and external validation across diverse populations therefore represent essential prerequisites for translating high-performing predictive models into reliable clinical tools [27].

Importantly, the clinical significance of AI extends beyond numerical accuracy. A clinically impactful model must improve decision-making, modify treatment planning, or enhance patient outcomes, not simply perform well on retrospective datasets. As emphasized in recent reviews, many AI systems lack evidence of real-world effectiveness, and their benefits remain largely theoretical until validated through prospective or multicenter studies [6,8]. Therefore, statistical performance should be viewed as a preliminary indicator, while clinical utility remains the ultimate benchmark for evaluating AI integration in implant dentistry.

Overall, bridging the gap between experimental results and clinical application requires comprehensive evaluation frameworks that incorporate interpretability, calibration, external validation, and prospective clinical impact analyses. Only through this multidimensional assessment can AI systems become reliable, ethically sound, and clinically meaningful tools for supporting implant treatment planning and long-term patient care [5].

### 5.6. External Validation, Dataset Quality, and Algorithmic Bias in AI-Driven Implant Dentistry

A major obstacle to the clinical deployment of AI systems in implant dentistry is the limited availability of robust external validation. Most published models are trained and tested on single-center datasets characterized by homogeneous demographic profiles, anatomical features, and imaging protocols. As reported in recent methodological analyses, monocentric models often show a significant decline in performance when applied to populations differing in age, ethnicity, bone morphology, or systemic conditions, raising concerns about their generalizability and reliability in broader clinical settings [14,28,29]. Demographic and anatomical bias represents an additional limitation. Craniofacial structures vary substantially across sex, age, and ethnic groups, influencing bone density and peri-implant anatomy. When specific populations are underrepresented, AI models may incorporate these disparities into their predictions, leading to skewed diagnostic or prognostic outputs, as widely documented in medical AI [30,31]. In implant dentistry, this may result in inaccurate risk estimation or misclassification of early pathological changes in underrepresented patient groups. Another important source of bias derives from the lack of standardization in imaging acquisition. Variability in CBCT and 2D radiographic parameters—including voxel size, exposure settings, field of view, and reconstruction algorithms—introduces noise that can compromise the performance of convolutional neural networks, which rely on pixel-level consistency for feature extraction [32]. Even minor differences in gray-value calibration or image resolution may generate predictions influenced by device-specific artifacts rather than true anatomical characteristics, thereby limiting cross-platform generalizability [33]. Algorithmic bias is further amplified by variability in diagnostic labeling and subjective interpretation. Definitions of conditions such as peri-implantitis or early marginal bone loss are not consistently applied across studies, and annotation quality depends on clinician expertise. Consequently, AI systems may learn statistical correlations rather than clinically meaningful patterns, potentially generating opaque or misleading predictions in real-world decision-making [9,34]. To address these challenges, rigorous external validation on independent, geographically and demographically diverse datasets is essential. In addition, calibration assessment is required to ensure that predicted risks accurately reflect observed outcomes across patient populations [27]. Transparent reporting of dataset composition, imaging protocols, and annotation methods, together with routine audits of algorithmic fairness, is increasingly recognized as a prerequisite for the responsible clinical implementation of AI systems [29,35]. Ensuring dataset diversity, standardized imaging workflows, and robust validation is therefore fundamental to developing AI tools that are safe, equitable, and trustworthy in implant dentistry.

## 6. Surgical AI Models for Planning, Navigation, and Execution

### 6.1. The Role of AI in Preoperative Implant Planning

Traditionally, implant planning was based on clinical assessment and two-dimensional radiography [36]. With the rise of digital dentistry, tools such as cone-beam computed tomography (CBCT), intraoral scanners, and 3D planning software have significantly improved the precision and predictability of implant placement. In this evolving digital framework, AI is emerging as a valuable adjunct, enhancing data analysis and enabling more personalized, precise, and efficient implant treatment planning. In detail, pre-surgical planning involves integrating data from CBCT, digital impressions, and facial scans into a single environment, requiring accurate anatomical segmentation—traditionally a time-consuming and expertise-driven task [37,38,39]. Automating the evaluation of bone quality and quantity on CBCT scans together with detection of critical anatomical structures (e.g., nerves and maxillary sinus, adjacent teeth) and prediction of drilling protocol has the potential not only to improve diagnostic accuracy but also to significantly reduce the risk of complications and overall time, effort, and costs associated with dental implant procedures. Moreover, AI enhances the design of surgical guides by providing highly accurate anatomical mapping, improving implant positioning, reducing complications, and supporting better clinical outcomes [40]. Several studies have explored AI’s role in clinical decision-making. Mangano et al. combined AI with augmented reality to plan guided implant surgery, reporting improved efficiency in straightforward cases [41]. Sakai et al. used AI models to predict drilling protocols from CBCT data with 93.7% accuracy, potentially supporting the estimation of primary stability [42]. About prosthetic planning, AI-based CAD software has been used to design final restorations that adapt to post-healing soft tissue contours. In a retrospective study, Lerner et al. applied AI-approach in 90 patients, demonstrating its feasibility for restoring 106 implant-supported zirconia crowns [43]. Nevertheless, its application is still limited. AI models show promise as decision support tools for surgical planning, offering capabilities in jawbone mineral density estimation, drilling protocol prediction, surgical classification for sinus augmentation, root inclination measurement, and implant-ridge relationship classification. Although some AI models outperform human experts in tasks, such as buccal bone thickness analysis, they remain in development and should complement expert judgments.

### 6.2. AI in Surgical Execution and Intraoperative Navigation

Beyond preoperative planning, AI is increasingly being incorporated into the intraoperative phase through real-time navigation systems and robot-assisted implant surgery. These technologies integrate AI-based image processing with dynamic tracking devices to enhance surgical accuracy, minimize deviation from planned trajectories, and reduce intraoperative complications [44]. Recent developments have enabled real-time comparison between the planned implant trajectory and the actual drilling path, providing immediate feedback to the surgeon [45]. This level of precision is particularly relevant in anatomically complex areas such as the posterior mandible or maxillary sinus region, where proximity to critical structures like the inferior alveolar nerve or maxillary sinus floor increases the risk of complications. AI-assisted navigation can also compensate for intraoperative variables, such as patient movement or soft tissue interference, ensuring consistent accuracy throughout the procedure [46]. Robot-assisted implant placement systems further leverage AI algorithms to refine handpiece motion, optimize drilling angulation, and reduce human error [47]. Several studies have demonstrated that AI-guided navigation may achieve comparable or superior accuracy compared to conventional guided surgery, with mean coronal deviations often below 1 mm and angular deviations under 3 degrees [48,49]. This integration has the potential to improve clinical predictability, minimize surgical time, and enhance patient safety.

## 7. Clinical Decision-Making Pathways for AI-Assisted Implant Dentistry

Although AI systems have demonstrated promising performance across diagnostic, prognostic, and surgical applications, their effective integration into clinical workflows requires a structured understanding of how algorithmic outputs should be interpreted and translated into care decisions. As emphasized in recent medical AI frameworks, algorithmic predictions should be considered supportive tools rather than autonomous decision-makers, reinforcing the central role of the clinician in interpreting model outputs within the broader clinical context [50]. For this reason, clinicians must critically evaluate AI predictions, contextualize them with patient-specific information, and remain aware of the limitations associated with model generalizability and dataset dependency [30].

In the diagnostic domain, AI-generated radiographic analyses can enhance early detection of peri-implant bone loss, radiolucency, and other subtle pathological alterations. Several studies have demonstrated the capacity of CNN models to improve diagnostic accuracy for early peri-implant disease by identifying features that may be overlooked during manual inspection [10,11,25,31]. However, these outputs should serve as an adjunctive “second opinion” rather than a definitive diagnostic conclusion. Clinical and radiographic assessment remains essential because AI systems may misclassify findings when confronted with atypical anatomy, image noise, or conditions underrepresented in their training data [31,51].

Similarly, prognostic models estimating long-term implant survival or failure risk offer valuable insights for personalized treatment planning. By integrating systemic, anatomical, and implant-specific variables, machine learning–based risk assessment tools can help clinicians identify high-risk scenarios and tailor preventive strategies accordingly [8,16,52]. Nevertheless, prognostic outputs must be interpreted cautiously. As highlighted by calibration studies in medical prediction modeling, AI-generated risk scores are probabilistic, may suffer from calibration drift, and often lack external validation across diverse populations [13,27]. Thus, such predictions should guide clinical planning without being regarded as deterministic indicators of future outcomes.

In the surgical context, AI contributes meaningfully to preoperative planning and intraoperative execution, enhancing workflow precision and reducing operator variability. Algorithms capable of segmenting anatomical structures, estimating bone density, and suggesting optimal implant trajectories have demonstrated potential benefits for improving predictability and safety in implant placement [41,42]. AI-assisted navigation and robotic platforms may reduce deviations from planned positions; however, intraoperative conditions frequently involve uncertainties that AI cannot anticipate—such as unexpected bleeding, irregular bone quality, or soft-tissue behavior. This reinforces that surgeon expertise and situational awareness remain central to safe surgical execution [32,53].

Overall, the integration of AI into implant dentistry should be conceptualized as a hybrid decision-making model, in which algorithmic insights enhance but never replace clinical reasoning. AI is most beneficial for tasks involving fine-grained pattern recognition, synthesis of large datasets, and standardization of repetitive procedures, while clinician judgment remains superior in scenarios requiring holistic assessment or nuanced interpretation of patient-specific factors. Establishing clear decision-making pathways is therefore essential to ensure that AI systems provide meaningful clinical value while maintaining the primacy of clinician oversight in treatment planning and execution.

## 8. Limitations and Future Perspectives

Although artificial intelligence (AI) has demonstrated significant promise in diagnostic and prognostic modeling—particularly in computer vision, image segmentation, and classification—several critical limitations persist [38]. These challenges mainly concern data quality and availability, limited model interpretability, restricted generalizability across heterogeneous populations, high implementation costs, and the need for robust computational infrastructure [54]. Moreover, clinical validation of many AI systems remains insufficient, representing a major barrier to their translation into routine practice. As a result, the reliability of AI models in predicting implant outcomes is not yet fully established, potentially leading to inappropriate patient assessment or suboptimal treatment planning when based on inaccurate virtual models [55,56]. A large proportion of existing studies rely on single-center datasets, substantially limiting external validity and reproducibility. The demographic and clinical homogeneity of these populations further restricts generalizability across diverse geographic and patient contexts. As highlighted by Schwendicke et al. [25], achieving true cross-center validity remains a major challenge for AI in dentistry, underscoring the need for large-scale, multicenter, and demographically diverse validation studies. While diagnostic models currently represent the most common AI application in dental research, prognostic modeling is emerging as an equally important field [27]. However, prognostic predictions cannot be immediately verified, making accurate calibration and clinician trust essential to avoid overtreatment or delayed intervention. Longitudinal studies with extended follow-up are therefore crucial to confirm long-term predictive validity and clinical relevance [27]. Beyond technical and methodological issues, ethical and regulatory aspects of AI implementation require careful consideration. Concerns related to data privacy, algorithmic bias, accountability, and transparency must be addressed to ensure ethical and equitable application in implant dentistry [57,58]. Accordingly, the establishment of standardized regulatory frameworks, validation protocols, and ethical guidelines is fundamental to guaranteeing safety and reliability. A scoping review by Elgarba et al. identified a substantial lack of rigorous clinical validation in automated implant planning software, emphasizing the need for randomized controlled trials involving large and diverse patient cohorts [59]. Future progress will depend on strengthening translational research, developing hybrid decision-support systems that integrate AI with clinician expertise, expanding the diversity of training datasets, and ensuring high-quality imaging for accurate anatomical modeling. Integration with complementary technologies such as augmented reality, digital twins, and intraoperative navigation may further enhance surgical precision and treatment planning [60]. Ultimately, multidisciplinary collaboration among clinicians, computer scientists, and biomedical engineers will be essential to translate AI research into clinically reliable, ethically grounded, and impactful tools.

## 9. Clinical Impact and Educational Perspectives

AI integration in implant dentistry not only reshapes clinical workflows but also transforms the educational landscape [61]. Virtual surgical simulators powered by AI can provide young clinicians with realistic and adaptive training environments, accelerating the acquisition of skills while minimizing patient risk [62]. Augmented reality (AR) and digital twins allow learners to rehearse complex implant procedures with immediate feedback, while AI algorithms adapt training scenarios based on individual performance metrics [63,64,65]. From a healthcare perspective, AI has the potential to reduce costs by optimizing resource allocation, shortening surgical time, and preventing complications. By supporting early diagnosis, risk stratification, and personalized follow-up, AI can contribute to more efficient and sustainable healthcare systems [66]. Therefore, its integration should be seen not only as a technological advancement but also as a strategic element in future dental education and health policy planning.

## 10. Conclusions

The literature reviewed indicates that AI can effectively support clinicians across diagnostic, prognostic, and operative stages; however, its integration into daily practice requires a clear understanding of both its capabilities and limitations. AI systems should be viewed as decision-support tools that enhance pattern recognition, risk stratification, and workflow standardization, rather than as replacements for clinical judgment. While AI may assist in identifying subtle radiographic changes and evaluating complex risk profiles, algorithmic outputs must always be interpreted within the broader clinical context of the individual patient. Current barriers to widespread clinical adoption include limited and demographically homogeneous datasets, variability in imaging protocols, inconsistent diagnostic labeling, and the limited use of explainable AI methods, all of which constrain generalizability and reliability. Future progress will depend on multicenter, prospective validation studies and the advancement of interpretable AI models aligned with real-world clinical needs. Ultimately, the value of AI in implant dentistry lies in augmentation rather than automation, supporting more predictable, personalized, and patient-centered implant care. This narrative review advances existing literature by shifting the focus from technical feasibility toward clinical integration, highlighting how AI outputs should be interpreted, contextualized, and applied within evidence-based implant dentistry.

## Data Availability

The original contributions presented in this study are included in the article. Further inquiries can be directed to the corresponding author.
